# Rapid Reduction of Phytotoxicity in Green Waste for Use as Peat Substitute: Optimization of Ammonium Incubation Process

**DOI:** 10.3390/plants13172360

**Published:** 2024-08-24

**Authors:** Wenzhong Cui, Juncheng Liu, Qi Bai, Lingyi Wu, Zhiyong Qi, Wanlai Zhou

**Affiliations:** 1 School of Mechanical Engineering, Chengdu University, Chengdu 610100, China; cuiwenzhong@stu.cdu.edu.cn (W.C.); liujuncheng@stu.cdu.edu.cn (J.L.); baiq_99@163.com (Q.B.); 82101225393@caas.cn (L.W.); 2 Chengdu National Agricultural Science and Technology Center, Institute of Urban Agriculture, Chinese Academy of Agricultural Sciences, Chengdu 610213, China; qizhiyong@caas.cn

**Keywords:** ammonium incubation, phytotoxicity, green waste, peat substitute

## Abstract

The rapid growth of the horticultural industry has increased demand for soilless cultivation substrates. Peat, valued for its physical and chemical properties, is widely used in soilless cultivation. However, peat is non-renewable, and over-extraction poses serious ecological risks. Therefore, sustainable alternatives are urgently needed. Ammonium incubation, a novel method to reduce phytotoxicity, offers the potential for green waste, a significant organic solid waste resource, to substitute peat. This study optimized the ammonium incubation process to reduce green waste phytotoxicity. It systematically examined different nitrogen salts (type and amount) and environmental conditions (temperature, aeration, duration) affecting detoxification efficiency. Results show a significant reduction in phytotoxicity with ammonium bicarbonate, carbonate, and sulfate, especially carbonate, at 1.5%. Optimal conditions were 30 °C for 5 days with regular aeration. Under these conditions, ammonium salt-treated green waste significantly reduced total phenolic content and stabilized germination index (GI) at a non-phytotoxic level (127%). Using treated green waste as a partial peat substitute in lettuce cultivation showed promising results. This low-cost, low-energy method effectively converts green waste into sustainable peat alternatives, promoting eco-friendly horticulture and environmental conservation.

## 1. Introduction

Due to the rapid growth of the horticultural industry, demand for soilless cultivation substrates has been increasing annually [1]. Peat is widely used as the most versatile plant growth medium due to its excellent aeration, water retention, buffering capacity, and nutrient supply capabilities [2,3]. However, peat is a non-renewable resource. Over-extraction can lead to biodiversity loss and excessive greenhouse gas emissions [4,5]. Therefore, finding sustainable alternatives to peat as plant growth media is urgently needed. Numerous studies have shown that green waste, after proper processing, exhibits favorable physicochemical properties and can serve as a substitute for peat in plant growth media compositions [6,7,8].

Green waste mainly refers to various plant residues and debris generated in landscaping, such as pruning branches and fallen leaves [9,10]. The volume of green waste is enormous; in China alone, it reached 40 million tons in 2018, with even higher quantities globally [11,12]. Currently, most green waste is disposed of through landfilling or incineration [13,14,15], causing environmental pollution and resource wastage [16,17]. Utilizing green waste as a substitute for peat in plant growth media not only reduces environmental pollution but also decreases the horticultural industry’s reliance on peat. However, green waste often possesses phytotoxicity due to substances like phenols and organic acids, which can inhibit plant growth. Therefore, it is crucial to remove or reduce its phytotoxicity before incorporating green waste into plant growth media [18]. The chemical constituents of green waste can dissolve into water and migrate to the plant root zone during application, Therefore, the removal of material phytotoxicity can be achieved by removing water-soluble phytotoxic substances from green wastes.

Ammonium incubation is an emerging technique for reducing phytotoxicity by rapidly decreasing the activity or concentration of phytotoxic substances in green waste. Previous studies on ammonium incubation involved mixing green waste and ammonium salts in a mass ratio of 1% to 2%, adjusting moisture content to 60% to 70%, and leaving them at room temperature for 5 days. This method significantly reduced phytotoxic substances and toxicity in green waste materials [19]. Compared to commonly used techniques like composting and heat treatment, ammonium incubation offers advantages such as low energy consumption, minimal pollution, simplicity, and low cost [5,19,20,21]. Additionally, it preserves the original volume and weight of green waste as much as possible [18]. Despite its benefits, ammonium incubation is a relatively new technology lacking comprehensive research and widespread application. This study also found significant variability in the effectiveness of ammonium incubation when applied to different types of green waste. Moreover, the incubation process likely involves complex chemical reactions and microbial activities. To enhance the detoxification efficiency and effectiveness of ammonium incubation, optimal environmental conditions for incubation need to be clearly defined.

This study systematically investigates the effects of detoxifying agents (types and amounts) and environmental conditions, including incubation temperature, aeration, and duration, on the effectiveness of ammonium incubation. The aim is to optimize key parameters of the ammonium incubation process and improve its ability to remove phytotoxicity from green waste effectively and consistently.

## 2. Materials and Methods

### 2.1. Materials

The green waste used in this study originated from street tree pruning waste in Changsha City, Hunan Province, China. Initially, the green waste was preliminarily crushed to a particle size of 1–2 cm, followed by drying at 85 °C for 24 h. Subsequently, a cutting mill (ST-R200, Xuxinshengke, Beijing, China) was used for further cutting and grinding, and the material was sieved through a 20-mesh screen (aperture size 0.9 mm). All processed green waste had particle sizes smaller than 0.9 mm. The bulk density of the waste material was 284 g/L, with a macroporosity of 5.02%, microporosity of 57.26%, and total porosity of 62.28%. The cellulose content was 20.51%, hemicellulose content was 18.47%, and lignin content was 32.41%.

### 2.2. Experimental Design

#### 2.2.1. The Effects of Detoxifiers

This study selected seven common nitrogen-containing compounds, including ammonium carbonate, ammonium sulfate, ammonium bicarbonate, ammonium chloride, ammonium dihydrogen phosphate, calcium nitrate, and potassium nitrate, to investigate their effects on the efficacy of detoxification treatments. Following the methodology of previous studies, green waste materials were mixed separately with (NH_4_)_2_CO_3_, (NH_4_)_2_SO_4_, NH_4_HCO_3_, NH_4_Cl, NH_4_H_2_PO_4_, Ca(NO_3_)_2_, and KNO_3_. The nitrogen application rate for each treatment was 2.92 mg·g^−1^ based on the dry weight of green waste (nitrogen quantity in compound/mass of green waste). The mixture was adjusted to 66.7% moisture content, thoroughly mixed, and then incubated at room temperature (22–30 °C) for 5 days [19]. Each incubation treatment was replicated three times.

To assess the detoxification effects of ammonium carbonate, four levels of ammonium salts were applied (0.5%, 1%, 1.5%, and 2% of ammonium carbonate relative to the dry weight of green waste). Specifically, 25 mg, 50 mg, 75 mg, and 100 mg of (NH_4_)_2_CO_3_ were added to 5 g of green waste, mixed thoroughly, adjusted to 66.7% moisture content, and then incubated at room temperature (22–30 °C) for 5 days. Each incubation treatment was replicated three times.

#### 2.2.2. The Effects of Environmental Conditions

**Figure 1:**
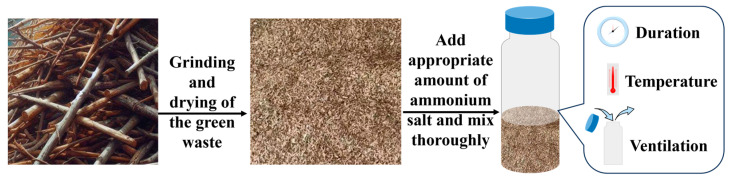
Ammonium incubation process and regulation of environmental conditions.

Environmental conditions were also evaluated using 1.5% (NH_4_)_2_CO_3_ (relative to the dry weight of green waste) as the detoxifying agent. Different incubation durations (1 day, 3 days, 5 days, 7 days, 9 days, 11 days) and temperatures (20 °C, 25 °C, 30 °C, 35 °C) were tested to explore their impact on the detoxification efficacy of ammonium salts. Ventilation conditions were varied with two treatments: sealed incubation and once-daily ventilation. Each incubation treatment was replicated three times (Figure 1).

#### 2.2.3. Cultivation Experiment of Green Waste as a Peat Substitute

For cultivation experiments replacing peat with detoxified green waste, a standard peat-based growing medium (CG) (peat:perlite:vermiculite = 9:3:1 *v*/*v*/*v*) was used as control. Two plant-growing media were prepared using shredded green waste to replace peat: the first using untreated green waste (OG) and the second using detoxified green waste under optimized ammonium incubation parameters (AG). In both media, green waste replaced 75% of peat by volume, while the proportions of perlite and vermiculite remained unchanged (green waste:peat:perlite:vermiculite = 6.75:2.25:3:1 *v*/*v*/*v*/*v*). The peat used in the study was moss peat (Pindstrup Sphagnum, Pindstrup, Denmark), size 10–30 mm. No fertilizers were added during the preparation of these growing media.

After seedlings were grown in seedling trays for 20 days, lettuce (Lactuca sativa, variety Rex Swan, obtained from Rijk Zwaan Export B.V., De Lier, The Netherlands) were transplanted into 500 cm^3^ plastic pots containing the aforementioned plant-growing media. Fifteen lettuce pots were randomly placed in a growth chamber (Sefo Experimental Instruments Co., Ltd., Ningbo, China) set to alternate between nighttime temperatures of 23 °C and daytime temperatures of 25 °C, with 67% humidity. Each lettuce pot received 100 mL of water every two days, with 100 mL of 50% Hoagland’s solution replacing water on the 6th, 12th, and 18th days after transplanting to supplement nutrients. Lettuce was harvested 28 days after transplanting.

### 2.3. Analytical Techniques

#### 2.3.1. Phytotoxicity Assessment

In this study, seed germination tests were used to assess the phytotoxicity of green wastes. *Brassica rapa chinensis* (Chinese cabbage) seeds, sensitive to phytotoxicity [22], were used for the germination tests. Water extracts were obtained following the classic method at a ratio of 1:10 (*w*/*v*). Specifically, 5 g of green waste samples were mixed with 50 mL of deionized water, shaken for 2 h, and centrifuged at 8000 rpm for 10 min at 4 °C. Subsequently, 10 mL of supernatant was collected and filtered through a 0.45 μm filter paper [23]. A 2 mL aliquot of the extract (with deionized water as control, denoted as CK for all control groups) was added to Petri dishes containing germination paper (diameter 9.0 cm), followed by the placement of 10 seeds on the germination paper. Each treatment’s extract underwent three replicate germination tests. Petri dishes were covered and sealed with parafilm to prevent moisture loss, then incubated in darkness at 25 °C for 72 h. The number of germinated seeds and the length of the radicle were recorded. Germination indices, including germination rate (GR), root length (RL), and germination index (GI), were calculated as follows [22]:

(1) $$GR\left(\%\right)=\frac{seed\;germination\;in\;treatment}{seed\;germination\;in\;control}*100\%$$

(2) $$\;RL\left(\%\right)=\frac{root\;length\;in\;treatment}{\;root\;length\;in\;control}*100\%$$

(3) $$GI\left(\%\right)=GR*RL$$

If the germination index (GI) is 80% or higher, the phytotoxicity of the material is considered extremely low, meeting the standard of harmlessness.

#### 2.3.2. pH, EC

The samples in this study were prepared using the same method as for the assessment of phytotoxicity experiments to obtain water extracts of green waste. pH and EC of the materials were measured using a multiparameter tester (Mettler-Toledo Instruments Ltd., Shanghai, China).

#### 2.3.3. Determination of Total Phenol

Total phenol in green waste materials was determined using a Plant Total Phenol Assay Kit (Boxbio, Beijing Bokai Biotechnology Co., Ltd., Beijing, China). According to the manufacturer’s instructions, the method is based on the reduction of tungsten molybdic acid to form a blue compound by phenolic substances under alkaline conditions. The product exhibits a characteristic absorption peak at 760 nm, and the total phenol content in plants is quantitatively detected by the change in absorbance. A 2 mL aliquot of water extract prepared before and after optimal ammonium incubation of green waste was tested following the kit’s instructions. The extraction method followed the procedures outlined in Section 2.3.1. Results are expressed in terms of gallic acid equivalents.

#### 2.3.4. Lettuce Biomass Measurement

Lettuce plants were harvested after 28 days of cultivation, and the above-ground parts were weighed fresh after clearing the growing medium. Subsequently, the samples are dried in an oven at 65 °C until a constant weight is achieved to determine the dry weight.

#### 2.3.5. OJIP Parameters Measurement

Chlorophyll fluorescence parameters of lettuce leaves were measured using a handheld chlorophyll fluorometer (FluorPen FP110, Photon Systems Instruments, Brno, Czech Republic). Due to higher physiological activity at the top leaves, which better reflects plant responses to environmental stressors, measurements were taken from the first fully developed leaf from the top left side, avoiding the main vein. Before measurement, leaves were dark-adapted using leaf clips for 30 min until reaching stable states. The rapid fluorescence rise from minimal to maximal fluorescence after dark adaptation was detected, with the instrument’s detector sensitivity exceeding 100,000 counts per second. Parameters interpretation and calculation formulas are detailed in Table A1 in Appendix A [24,25].

### 2.4. Statistical Analyses

Each treatment in the cultivation experiment was replicated 5 times, and other treatments and analyses were replicated three times each. Data are presented as mean ± standard deviation. Statistical analysis of the phytotoxicity assessment experiment was performed using SPSS 27. One-way ANOVA and Tukey’s post hoc test were used to explain the differences in the measured parameters in the cultivation experiment.

## 3. Results and Discussion

### 3.1. Effects of Detoxifying Agent Types and Dosages on Detoxification Efficacy

**Figure 2:**
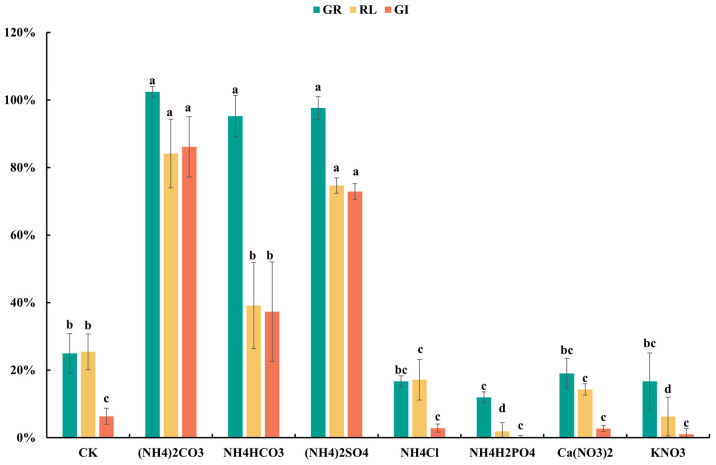
GR, RL, and GI of green wastes before (CK) and after incubation with different ammonium salts and nitrates. Error bars represent standard deviation (*n* = 3). Within each germination index, values that do not share a letter differ significantly according to ANOVA and Duncan’s multiple range test (*p* < 0.05).

**Figure A1:**
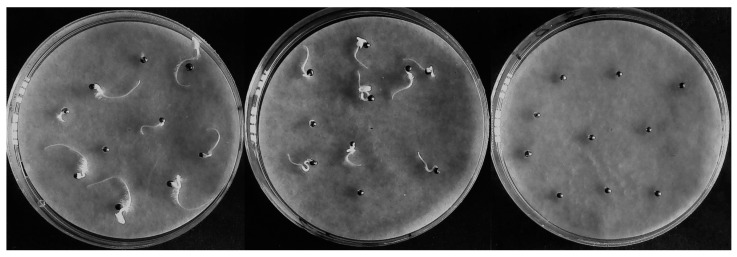
Seed germination at 36 h (from left to right for (NH_4_)_2_CO_3_, (NH_4_)_2_SO_4_, NH_4_HCO_3_ treatment groups).

Ammonium salts significantly reduce the phytotoxicity of materials, but their effectiveness varies greatly depending on the type of ammonium salt used. As shown in Figure 2, water extracts from raw garden waste materials markedly inhibited seed germination and root growth, indicating high phytotoxicity. Incubation with (NH_4_)_2_CO_3_, NH_4_HCO_3_, and (NH_4_)_2_SO_4_ significantly increased seed germination rates (GR) from 25% to 102%, 95%, and 98%, respectively, and ultimately elevated the seed germination index (GI) from 6.4% to 86.1%, 37.3% and 72.9%, demonstrating a significant reduction in the phytotoxicity of green waste. Additionally, compared to incubation with (NH_4_)_2_CO_3_ and (NH_4_)_2_SO_4_, seed germination was delayed after incubation with NH_4_HCO_3_ (Figure A1). This delay in germination may shorten the period for root elongation during germination tests, resulting in reduced root lengths and correspondingly lower GI [26].

Nitrogen has a profound impact on plant growth and development by participating in protein synthesis and influencing overall plant growth and development [27,28]. Ammoniacal and nitrate nitrogen are readily soluble in water, serving as quickly available nutrients for plants. To investigate the nutrient effects on seed germination promotion, different forms of nitrogen incubation experiments were conducted. As shown in Figure 2, incubation with Ca(NO_3_)_2_ and KNO_3_ did not promote seed germination of green waste seeds, with germination rates (GR) of only 19% and 17% and final seed germination indices (GI) of 2.7% and 1.0%, respectively, indicating that nitrogen in nitrate form does not effectively promote seed germination and growth. Furthermore, even with ammoniacal nitrogen, incubation with NH_4_Cl and NH_4_H_2_PO_4_ did not increase but rather decreased the seed germination index of green waste, with GI values of 2.9% and 0.2%, respectively. Seed germination was severely inhibited in these incubation treatments, indicating that only appropriate ammonium salts such as (NH_4_)_2_CO_3_, NH_4_HCO_3_, and (NH_4_)_2_SO_4_ can effectively reduce or eliminate the phytotoxicity of green waste. Among these, (NH_4_)_2_CO_3_ showed the most optimal detoxification effect.

**Figure 3:**
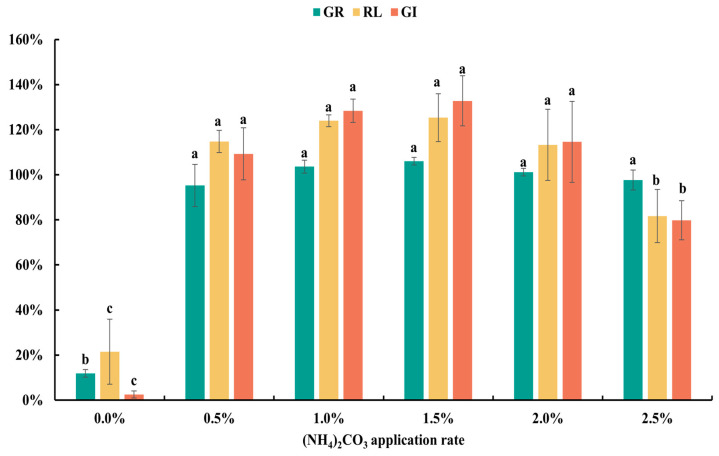
GR, RL, and GI of green waste treated by (NH_4_)_2_CO_3_ incubation at different dosages. Error bars represent standard deviation (*n* = 3). Within each germination index, values that do not share a letter differ significantly according to ANOVA and Duncan’s multiple range test (*p* < 0.05).

For plants, ammonium ions serve as a rapid nitrogen source, but the previous research found that concentrations exceeding 1 mg·mL^−1^ significantly inhibit seed germination, indicating high levels of phytotoxicity [19]. Therefore, it is crucial to control the dosage of ammonium salts during incubation to ensure effective detoxification of materials without introducing new phytotoxic substances [29]. As shown in Figure 3, this study applied 0.5% to 2.0% (NH_4_)_2_CO_3_ for incubating green waste materials. Seed germination in water extracts was normal across all concentrations, with GR reaching 95%, 104%, 106%, and 98%, respectively, and GI significantly increased, achieving 109.3%, 128.4%, 132.8%, and 79.7%. Except for the 2.5% (NH_4_)_2_CO_3_ experimental group, all reached the non-phytotoxic standard (GI ≥ 80%), with the 1.5% (NH_4_)_2_CO_3_ dosage demonstrating optimal detoxification effects, achieving a GI of 132.8%.

The variability in detoxification effects due to different ammonium salt dosages can be attributed to the dual role of ammonium carbonate. On the one hand, as a detoxifying agent, (NH_4_)_2_CO_3_ may react with major plant phytotoxic substances such as organic acids and phenols, therefore reducing their content; insufficient dosage may lead to incomplete removal of toxic substances. On the other hand, excessive application of (NH_4_)_2_CO_3_ can increase ammonia concentrations, which themselves can induce phytotoxicity. Therefore, achieving the optimal balance in the application of detoxifying agents is crucial for effective detoxification without introducing additional phytotoxicity. From a production cost perspective, a 0.5% application rate of the detoxifying agent may be more appropriate. Materials treated at this rate still meet non-phytotoxic standards and can significantly reduce production costs.

### 3.2. Effects of Environmental Conditions on Detoxification Efficacy

**Figure 4:**
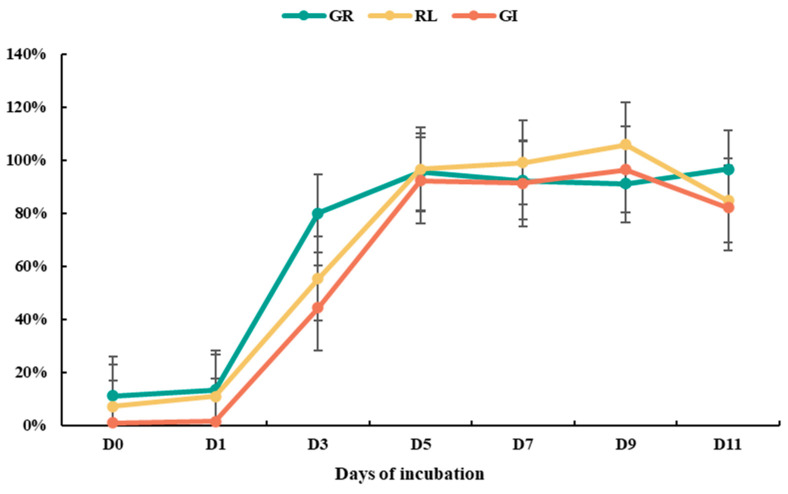
GR, RL, and GI of green wastes treated by ammonium incubation for different days. Error bars represent standard deviation (*n* = 3).

As shown in Figure 4, during the incubation of green waste with ammonium salts, there was little change in GI after 1 day, remaining close to 0% similar to the original material. After 1 day of incubation, green waste still significantly inhibited seed germination, with no significant difference in germination rate (GR) compared to the original material. However, by day 3 of incubation, GI rapidly increased to 44.3%, and unlike the 1-day incubation, the material no longer significantly inhibited seed germination, with GR reaching 80%. By day 5, GR of green waste increased to 96%, relative root length (RL) significantly increased to 97% compared to the 3-day incubation group, and GI reached 92%. From day 5 to 9 of incubation, GI remained stable between 91.3% and 96.4%. However, after 9 days of incubation, there was a decline in GI, reaching 82% by day 11. Although this experimental group did not inhibit seed germination, GR remained at a high level of 97%, whereas RL decreased to 85% compared to the 5–9-day incubation groups. Considering time constraints, a 5-day incubation period appears ideal.

Previous studies suggested that during ammonium incubation, ammonium ions react chemically with acidic phenols and organic acids in green waste, reducing the content of phytotoxic substances and, therefore, lowering the material’s phytotoxicity [19]. However, chemical reactions typically occur quickly. In this study, the significant reduction in phytotoxicity of green waste after 3 days of ammonium incubation suggests that the detoxification process may involve substance transformations possibly mediated by microbial activity rather than solely chemical reactions.

**Figure 5:**
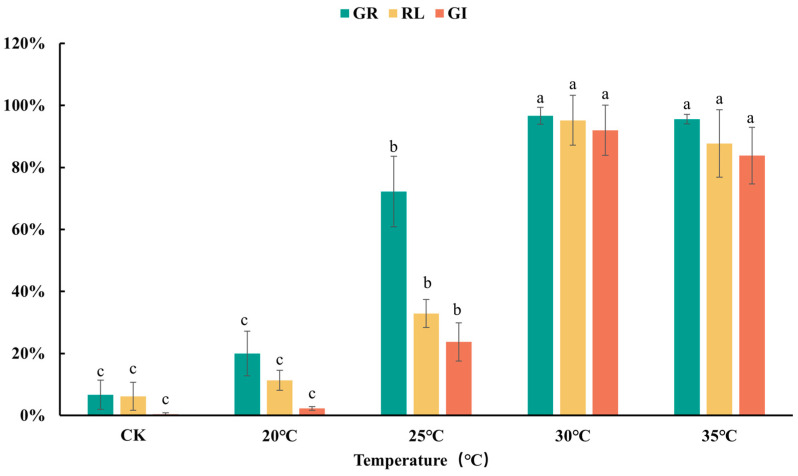
GR, RL, and GI of green waste treated by ammonium incubation at different temperatures. CK represents untreated green waste. Error bars represent standard deviation (*n* = 3). Within each germination index, values that do not share a letter differ significantly according to ANOVA and Duncan’s multiple range test (*p* < 0.05).

Temperature also significantly affects the detoxification efficiency of ammonium incubation, as shown in Figure 5. GI of green waste after incubation increased with temperature, reaching 2.3% and 23.7% at 20 °C and 25 °C, respectively. At 30 °C, GI peaked at 92%, slightly decreasing to 83.8% at 35 °C. Incubation at 20 °C still inhibited seed germination, with GR only at 20%. At 25 °C, the inhibitory effect on seed germination was significantly reduced (72% GR), but root growth was minimal (RL 33%), resulting in a GI of 23.7%. Incubation at 30 °C and 35 °C did not inhibit seed germination, with GR reaching 97% and 96%, respectively. However, at 35 °C, RL decreased from 95% to 88%. These results indicate that around 30 °C, ammonium incubation achieves optimal detoxification, making it suitable for the harmless treatment of garden waste materials. The optimal temperature range aligns well with the growth conditions of mesophilic microorganisms, whose optimal proliferation temperatures are typically around 30 °C [30,31], further suggesting their critical role in the detoxification process.

**Figure 6:**
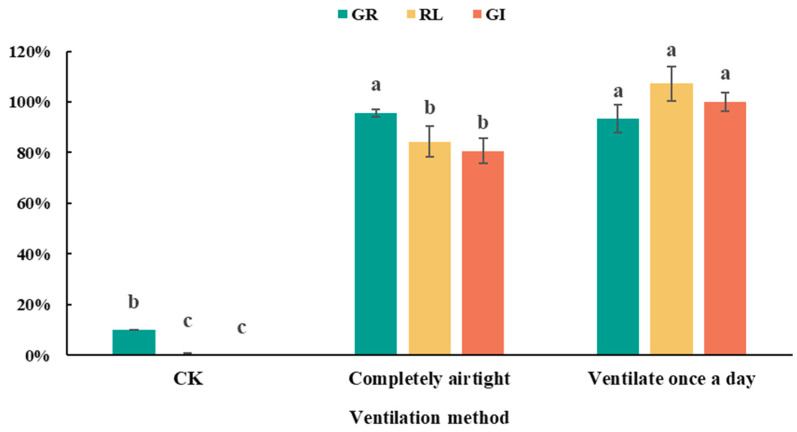
GR, RL, and GI of green waste treated by ammonium incubation at different aeration conditions. CK represents untreated green waste. Error bars represent standard deviation (*n* = 3). Within each germination index, values that do not share a letter differ significantly according to ANOVA and Duncan’s multiple range test (*p* < 0.05).

Similarly, different ventilation conditions during incubation also resulted in significant differences in detoxification effects, as shown in Figure 6. Both hermetic and once-daily ventilated incubation significantly reduced the phytotoxicity of green waste compared to the original material, with GI reaching 80.5% and 100%, respectively. The difference in GI levels between the two treatments was primarily due to differences in root length growth, with RL values of 84% and 107%, respectively. However, both treatments exceeded the non-toxic standard (GI ≥ 80%), indicating that proper ventilation conditions can significantly enhance the detoxification effect. Previous studies indicated that water-soluble substances such as organic acids and amino acids are major contributors to the phytotoxicity of green waste, and reducing their content can significantly decrease the material’s phytotoxicity [32]. Composting studies with agricultural waste have shown that water-soluble organic compounds like organic acids and phenolic acids can be utilized as carbon sources by microbes, leading to their decomposition [33,34,35]. However, microbial decomposition typically requires suitable C/N ratios, pH, and temperature conditions, with green waste composed of plant residues and debris typically having high C/N ratios [36]. During ammonium incubation, the addition of ammonium salts can lower the material’s C/N ratio, potentially enhancing microbial activity and promoting the degradation of water-soluble phytotoxic substances such as organic acids and amino acids, therefore reducing phytotoxicity. The detoxification effect of ammonium incubation was evident only after 3 days, similar to the duration required for rapid degradation of soluble substances observed in many composting studies [37,38], further supporting this hypothesis.

Temperature and oxygen content are key factors influencing the activity of aerobic microorganisms. In this study, optimal detoxification effects of ammonium incubation were observed at around 30 °C under appropriate ventilation conditions, suggesting a critical role for microbial processes in ammonium salt-induced detoxification. 

It is noteworthy that while nitrates can also serve as nitrogen sources for microbes and reduce the C/N ratio of materials, this study found that nitrate incubation did not reduce the phytotoxicity of green waste. This may be due to conditions in woody medium environments rich in ammonium salts inducing and promoting nitrification processes [39]. Nitrifying bacteria thrive in neutral to slightly alkaline environments (pH 7.0–8.2) at around 30 °C and require dissolved oxygen levels of 2–5 mg/L for optimal growth and activity. Lack of oxygen or low oxygen content is detrimental to the growth and activity of nitrifying bacteria, conditions which are consistent with the optimal ammonium incubation conditions identified in this study, further indicating the pivotal role of nitrification microbial activity in determining the detoxification effect of ammonium incubation [40,41,42,43]. Future research should investigate changes in microbial communities during ammonium incubation to confirm these hypotheses.

### 3.3. Verification of Detoxification Effects of Ammonium Incubation

**Figure 7:**
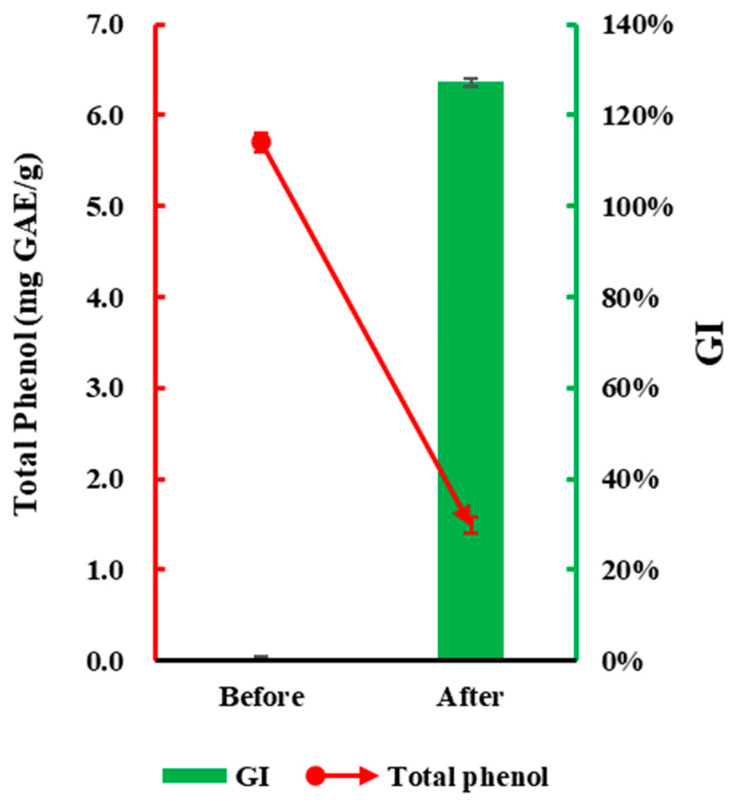
Total phenol content and phytotoxicity of green waste before and after ammonium incubation under optimal conditions. Error bars represent standard deviation (*n* = 3).

A wealth of studies indicates that phenolic compounds are the primary source of phytotoxicity in green waste, making changes in phenolic content a key indicator of phytotoxicity changes [32,34,44,45]. As shown in Figure 7, under the optimized conditions determined in this study (1.5% ammonium carbonate addition, 5 days of incubation at 30 °C with daily ventilation), there was a significant difference in total phenol content of green waste before and after ammonium incubation. The original material had a total phenol content of 5.70 mg·g^−1^, whereas, after ammonium incubation, the total phenol content of green waste significantly decreased to 1.49 mg·g^−1^, indicating a significant reduction in phytotoxic substances. The change in GI of green waste before and after ammonium incubation, ranging from 0% to 127%, also indicates that after ammonium salt incubation treatment, the phytotoxicity of green waste has been effectively eliminated.

Furthermore, cultivation substrates with phytotoxicity essentially impose stress conditions that significantly affect the plant photosynthesis process. Analysis of chlorophyll fluorescence dynamics provides detailed information about the photosynthetic apparatus, particularly the structure and function of PSII. In this study, we compared the effects of ammonium incubation detoxification treatment (AG) and untreated (OG) horticultural waste substrates on plant chlorophyll fluorescence parameters to assess the mitigating effect of ammonium incubation treatment on phytotoxicity. Table 1 shows that ϕPo (maximum photochemical quantum yield) of AG treatment was 0.83 ± 0.01, while for OG treatment, it was only 0.79 ± 0.02, indicating that ammonium incubation treatment significantly enhanced the photochemical efficiency of PSII, implying that more light energy was effectively used in the photosynthesis process rather than being lost due to damage to the photosystem [46]. In addition, ϕEo (electron transport quantum yield) and ϕRo (total electron transport quantum yield) of AG treatment were also significantly higher than those of OG treatment, indicating that the substrate treated with ammonium incubation not only improved the efficiency of electron transfer from QA- to QB but also enhanced the overall functionality of the electron transport chain, therefore improving the performance of Photosystem II. δRo (electron transfer efficiency from QA- to PQ) was also significantly higher in the AG treatment group than in the OG treatment group (0.16 ± 0.01 vs. 0.13 ± 0.06), further indicating that ammonium incubation treatment effectively reduced the damage to Photosystem II [47].

Performance index (PI) is a parameter that comprehensively reflects the absorption, capture, and electron transfer capabilities of Photosystem II. The PI of the AG treatment group was 4.80 ± 0.66, significantly higher than the 2.24 ± 0.92 of the OG treatment group, indicating that ammonium incubation treatment significantly improved the overall performance of Photosystem II. The significant increase in PI implies that plants in the substrate after detoxification treatment can perform photosynthesis more efficiently, therefore alleviating the stress caused by the phytotoxicity of green waste substrate. These results indicate that after ammonium incubation detoxification treatment, the substrate of green waste significantly reduces stress on lettuce, manifested by significantly enhanced photochemical and electron transfer efficiency of Photosystem II, reduced damage to the photosystem, and thus increased photosynthetic capacity. (Refer to Table A1 in Appendix A for an explanation of the chlorophyll fluorescence parameters).

Throughout the cultivation process, lettuce grown in AG (ammonium salt-treated) substrate exhibits full and lush foliage, with leaves displaying the normal purple-green color characteristic of this lettuce variety (Monde, a lettuce field variety from Rijk Zwaan Export Company, De Lier, The Netherlands). In contrast, lettuce grown in OG (untreated) substrate has significantly fewer leaves, and after 28 days of transplanting, the leaves appear yellowish. Additionally, the number and size of lettuce leaves in OG substrate show minimal change between 14 and 28 days after transplanting, indicating a significant slowdown in growth rate after 14 days, reflecting strong phytotoxicity of the OG substrate. On the other hand, lettuce grown in AG substrate, although with lower biomass compared to CG (peat-treated) substrate, exhibits normal growth throughout the growth cycle with good vigor, showing no apparent phytotoxic effects.

**Figure 8:**
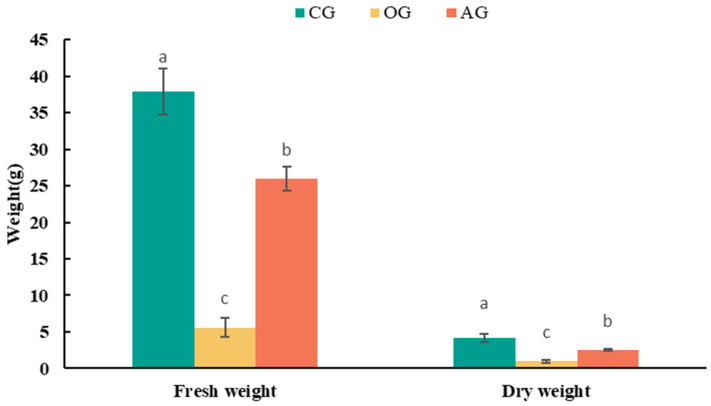
Biomass of lettuce grown in different growing media. Error bars represent standard deviation (*n* = 3). Within each index, values that do not share a letter differ significantly according to ANOVA and Duncan’s multiple range test (*p* < 0.05).

As depicted in Figure 8, compared to the untreated green waste substrate (OG), lettuce grown in the detoxified green waste substrate (AG) shows a significant increase in individual plant biomass. Fresh weight per plant increased markedly from 5.60 g to 24.94 g, while dry weight per plant increased from 0.96 g to 2.39 g. This correlates with the earlier findings of chlorophyll fluorescence parameters, indicating that after ammonium incubation detoxification treatment, the phytotoxicity of green waste substrate is significantly reduced, alleviating stress on crops, enhancing photosynthetic efficiency, and ultimately showing improved growth performance.

Figure 8 also indicates that the biomass of lettuce grown in ammonium salt-treated detoxified green waste substrate remains slightly lower than that in peat control treatment, possibly due to the significant influence of air, water, and nutrient conditions on plant growth in the absence of phytotoxicity. As a soilless cultivation medium, substrate porosity and water retention capacity affect air, water exchange, and nutrient absorption during plant growth [48,49]. As shown in Table 2, using green waste instead of peat as a plant growth substrate shows significant differences in porosity and water retention compared to traditional peat substrates, with higher water retention pore space (close to 70%) and lower aeration pore space (less than 5%), which may lead to inadequate root aeration for lettuce, potentially explaining why the growth performance of lettuce in green waste substrate is inferior to that in peat.

## 4. Conclusions

This study systematically investigates the effects of different detoxification agents (types and concentrations) and environmental conditions (including incubation temperature, aeration, and treatment duration) on the detoxification of ammonium salt-treated green waste. Experimental results demonstrate that under appropriate concentrations and incubation conditions, ammonium carbonate significantly reduces the phytotoxicity of green waste. Specifically, treating with 1.5% ammonium carbonate under adequate aeration at 30 °C for five days markedly reduces the total phenol content of the green waste and effectively mitigates its phytotoxic effects on plants.

Furthermore, this study explores the feasibility of using detoxified green waste as a partial substitute for peat in preparing plant-growing media. It was found that untreated original green waste exhibits strong phytotoxicity, leading to significant growth inhibition when used directly as a hydroponic substrate. In contrast, after ammonium incubation, the phytotoxicity of green waste is significantly reduced, alleviating stress on crops and resulting in significantly improved plant growth performance compared to untreated waste.

Ammonium incubation emerges as a promising new technology due to its low energy consumption, lack of pollution, simplicity of operation, and low cost. Compared to traditional composting methods, this technique requires less time and minimally alters the volume of raw materials, which is crucial for the effective utilization of agricultural waste and potential enhancement of production efficiency. Future research could further explore the application of ammonium incubation technology in handling garden waste from different countries and regions, therefore promoting its large-scale application in practical production.

## Figures and Tables

**Table 1:** Chlorophyll fluorescence parameters of lettuce grown in different growing media.

Growing Media	ϕPo	ϕEo	δRo	ϕRo	PI_abs_
AG	0.83 ± 0.01	0.50 ± 0.02	0.16 ± 0.01	0.08 ± 0.01	4.80 ± 0.66
OG	0.79 ± 0.02 *	0.44 ± 0.03 **	0.13 ± 0.06 *	0.06 ± 0.03 *	2.24 ± 0.92 **

*, ** represent differences at *p* < 0.05 and *p* < 0.01 levels according to *t*-test, respectively.

**Table 2:** Basic physicochemical properties of different growing media.

Growing Media	BD (g/cm^3^)	TP	WHC	AFP
CG	0.172 ± 0.006 ^b^	65.0% ± 4.1% ^b^	57.0% ± 4.5% ^b^	8.0% ± 0.5% ^a^
AG	0.260 ± 0.013 ^a^	73.7% ± 3.2% ^a^	69.0% ± 2.4% ^a^	4.7% ± 1.1% ^b^
OG	0.259 ± 0.002 ^a^	70.2% ± 1.4% ^ab^	68.1% ± 1.5% ^a^	2.1% ± 0.1% ^c^

TP represents total porosity; AFP represents air-filled porosity; WHC represents water-holding capacity; BD represents bulk density. Within each index, values that do not share a letter differ significantly according to ANOVA and Duncan’s multiple range test (*p* < 0.05).

## Data Availability

Data are contained within the article.

## Appendix A

**Table A1:** Interpretation and calculation of indicators for the determination of chlorophyll fluorescence parameters.

Calculation Formulas	Parameters Interpretation
FO=F(20μs)	F0 (Minimum Fluorescence): In the state of dark adaptation, when all reaction centers are open, i.e., the primary electron acceptor QA in the reaction center is in an oxidized state, the fluorescence value is observed. This reflects the baseline fluorescence of the reaction center of Photosystem II (PSII), mainly originating from antenna pigments.
FM=FP=F(t)max	FM (Maximum Fluorescence): The fluorescence value when all reaction centers are closed in the state of dark adaptation. This reflects the fluorescence of antenna pigments after maximum photochemical quenching.
FV=FM−FO	
ABS/RC=[4(F300μs−FO)/(F2ms−FO)](FM/FV)=M0(1/VJ)(1/ϕPo)	Energy flux absorbed by the active reaction center of PSII per unit.
M0=(ΔV/Δt)0=4(F300μs−FO)/(FM−FO)=TR0/RC−ET0/RC	The approximate initial slope (per millisecond) of transient fluorescence V = f(t). This parameter reflects the closing rate of the active reaction centers (RCs) of PSII.
TR0/RC=ϕPo(ABS/RC)	Energy flux captured by the active reaction center of PSII per unit at t = 0
ET0/RC=ϕEo(ABS/RC)	Energy flux transmitted by the active reaction center of PSII per unit at t = 0
RE0/RC=ϕRo(ABS/RC)=(1−VI)×(M0/VJ)	At t = 0, the ratio of electron flux reducing the terminal electron acceptor of PSⅠ per active reaction center of PS.Ⅱ
DI0/RC=(ABS/RC)−(TR0/RC)	At t = 0, the energy flux dissipated as heat per active reaction center of PSII.
ϕPo=TR0/ABS=FV/FM=1−FO/FM	At t = 0, the maximum quantum yield of the primary photochemical reaction, which refers to the quantum yield of photon capture by the PSII reaction center, also represents the maximum photochemical efficiency of the PSII reaction center in the state of dark adaptation.
ψEo=ET0/TR0=(1−VJ)	At t = 0, the ratio of electrons captured by the active reaction center of PSII that are transferred.
ϕEo=ET0/ABS=(1−FO/FM) ψEo=ϕPoψEo	At t = 0, the quantum efficiency of electrons transferred from QA- to the electron transport chain.
ψRo=RE0/TR0=1−VI	At t = 0, the ratio of electrons captured by the active reaction center of PSII that are transferred to PSI.
δRo=RE0/ET0=(1−VI)/(1–VJ)	The ratio of electrons reducing the terminal electron acceptor of PSI to the electrons in the electron transport chain.
ϕRo=RE0/ABS=ϕPoψRo=ϕPoψEoδRo	Quantum yield of reducing the terminal electron acceptor on the acceptor side of PSI.
ϕDo=FO/FM=1−ϕPo	At t = 0, the quantum yield of heat dissipation reflects the proportion of absorbed light energy in Photosystem II (PSII) that is dissipated by the non-photochemical quenching process.
PIabs=[γo/(1−γo)][ ϕPo/(1−ϕPo)][ψEo/(1−ψEo)]	Performance index based on absorption: It describes the performance index from the absorption of photons to the reduction of the electron transport chain.
